# The Impact of Experimental Conditions on Cell Mechanics as Measured with Nanoindentation

**DOI:** 10.3390/nano13071190

**Published:** 2023-03-27

**Authors:** Martina Zambito, Federica Viti, Alessia G. Bosio, Isabella Ceccherini, Tullio Florio, Massimo Vassalli

**Affiliations:** 1 Dipartimento Medicina Interna, Sezione di Farmacologia, Università di Genova, viale Benedetto XV 2, 16132 Genova, Italy; 2 Institute of Biophysics, National Research Council, Via De Marini 6, 16149 Genova, Italy; 3 IRCCS Istituto Giannina Gaslini, 16147 Genova, Italy; 4 IRCCS Ospedale Policlinico San Martino, Largo Rosanna Benzi 10, 16132 Genova, Italy; 5 James Watt School of Engineering, University of Glasgow, Glasgow G12 8QQ, UK

**Keywords:** nanoindentation, elasticity, cell culture

## Abstract

The evaluation of cell elasticity is becoming increasingly significant, since it is now known that it impacts physiological mechanisms, such as stem cell differentiation and embryogenesis, as well as pathological processes, such as cancer invasiveness and endothelial senescence. However, the results of single-cell mechanical measurements vary considerably, not only due to systematic instrumental errors but also due to the dynamic and non-homogenous nature of the sample. In this work, relying on Chiaro nanoindenter (Optics11Life), we characterized in depth the nanoindentation experimental procedure, in order to highlight whether and how experimental conditions could affect measurements of living cell stiffness. We demonstrated that the procedure can be quite insensitive to technical replicates and that several biological conditions, such as cell confluency, starvation and passage, significantly impact the results. Experiments should be designed to maximally avoid inhomogeneous scenarios to avoid divergences in the measured phenotype.

## 1. Introduction

In the last few decades, the mechanical properties of single cells have emerged as an important phenotypic trait to understand key physiological mechanisms, such as stem cell differentiation [1] and embryogenesis [2], as well as pathological processes such as cancer invasiveness [3] and endothelial senescence [4]. The viscoelastic properties of the cytoskeleton and the nucleus are intimately linked to mechanotransduction [5], influencing the ability of cells to sense their microenvironment and adapt to the extracellular matrix [6]. The mechanical properties of cells are a direct proxy of the biological state and constitute a very promising physio-pathological biomarker [7].

The de facto standard for measuring single-cell mechanics is nanoindentation [8], either using an Atomic Force Microscope (AFM) [9], eventually with a colloidal probe [10], or more dedicated devices with different deflection detection methods [11]. The experimental procedure to perform nanoindentation and analyze the data is well established [12]. Nevertheless, obtaining robust and reproducible mechanical characterization of single cells is still a challenging task. Living cells are complex systems and the description of their mechanical properties in terms of a single modulus largely depends on the methods used to probe them, with results that can vary by up to 1000 times depending on the cell type and experimental technique [13]. Even when the same methodology is used, the results still vary and are poorly comparable across different groups due to calibration issues [14] and the impact of the analysis pipeline on the final results [15]. For this reason, many groups have suggested, wherever possible, evaluating relative changes in the mechanical properties, in an attempt to cancel out all major sources of instrumental errors [16,17]. However, the variability of the results of single-cell mechanical measurements not only depends on systematic instrumental errors but is also impacted by the experimental design and sample conditions (sample replication, cell passage, shape, etc.). In this paper, we explored the impact of several experimental parameters associated with the indentation of living cells to highlight the most critical aspects to be taken into account to improve the repeatability and reliability of nanoindentation-based single cell mechanical characterization.

## 2. Materials and Methods

### 2.1. Experimental Design

**Figure 1:**
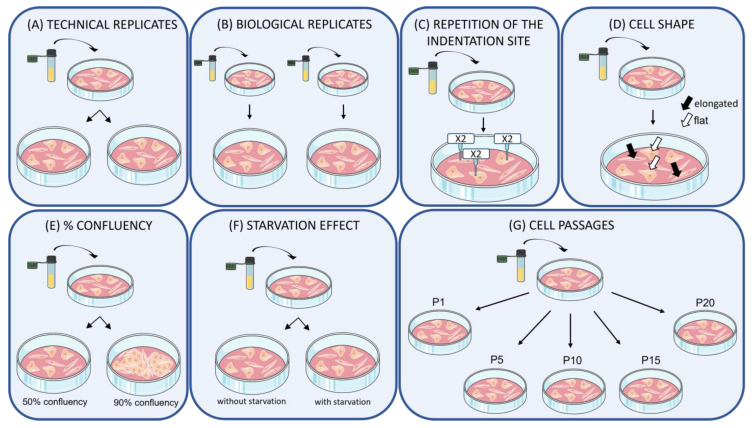
Assessment of cell elasticity values under different conditions and experimental designs.

Figure 1 presents a sketch of the main conditions that were evaluated in this work.

Cell elasticity values have been compared under different experimental conditions, sketched in Figure 1: (A,B) the comparison of elasticity data obtained over technical replicates, as well as those retrieved from biological replicates; (C) the maintenance of stiffness values when indentation occurs repeatedly in the same indentation cell site; (D) the evaluation of stiffness obtained from indenting cells showing different shapes; (E) the measure of elasticity of cells at different levels of cell confluence; (F) the evaluation of stiffness values in cells with or without starvation; (G) possible changes in elasticity during cell aging (considered as cell passages). These conditions are often considered in experimental design, and as critical features for stiffness measurement, could influence the robustness of the retrieved elasticity data. To test this hypothesis, identical cell culture steps (see Section 2.2), acquisition modes, and data analyses (see Section 2.3) were carried out, while enabling the listed conditions, to verify the steadiness and reproducibility of cell elasticity results.

### 2.2. Samples and Culture Treatments

Primary skin fibroblast cell lines were provided by Institute Giannina Gaslini in Genova (Italy). They were collected and made available from their Biobank service. Seven lines were used, from either healthy individuals (lines 4 and 5) or patients affected by pathologies, such as intestinal (lines 1, 3, 6, 7), and urogenital (line 2). The choice of relying on different fibroblast lines, presenting individual peculiarities, aims at showing nanoindentation reproducibility in the presence of genetic variability of the same primary cell type. Fibroblasts were cultured in RPMI medium, supplemented with 10% fetal bovine serum, PenStrep 1%, and glutamine 1%, and were maintained at 37 °C in a 5% CO_2_ incubator in a humidified atmosphere. In all tests, except for those involving cell aging, cells were evaluated at similar passages (between passages 3 and 5). Except for tests on the effect of starvation, in order to obtain homogeneous performances over the whole cell population, a synchronization protocol was applied consisting of total serum depletion on adherent, non-confluent cells for 48 h. To this end, cells were washed first in PBS, then in PBS-albumin 3%, and finally again in PBS before being plated in culture medium w/o FBS. The experiments were performed at room temperature, so the serum-free medium was replaced and supplemented with HEPES, in order to stabilize the pH. Each experimental test did not take more than one hour per plate.

### 2.3. Cell Indentation and Data Analysis

**Figure 2:**
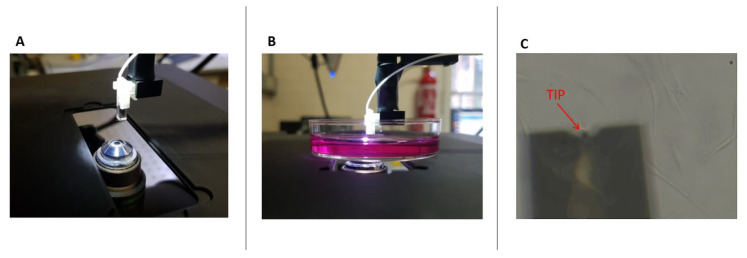
Indentation setup. (**A**) Alignment of the microscope objective with the nanoindentation probe. (**B**) Sample on the objective, during an indentation experiment. (**C**) Phase contrast image of the cantilever approaching a cell layer.

Single-cell stiffness measurements were performed using a Chiaro system (Optics11Life, Amsterdam, The Netherlands), a nanoindentation device based on a ferrule-top with interferometric read-out that enables high-resolution force measurements in a liquid environment [11]. The experimental protocol to operate the Chiaro nanoindenter is very similar to that required for the more widely adopted atomic force microscope (AFM), and the data can be analysed using the same approach [11,12]. In brief, the force *F* is measured while moving the tip towards the sample (along the vertical axis, *Z*) and the corresponding F(Z) curve is recorded. The Chiaro device is mounted on a holographic microscope (HoloMonitor 3, Phase Holographic Imaging, PHI AB, Lund, Sweden) with phase-contrast mode that allows us to precisely target individual cells with the tip (Figure 2). Experiments were carried out with a constant approach speed of 2.5 µm/s using a soft cantilever (stiffness 0.025 N/m) and a spherical tip with a radius R of 3 µm to avoid cell damage and to guarantee a definite contact area. Under these conditions, the indentation process is minimally invasive for the cell, causing no significant alteration in its morphology. The mechanical properties of the cells were calculated by fitting the indentation curve with the Hertz model ([18,19]), considering most often an indentation depth of 300 nm, selected to be smaller than 10% of the thickness of the cell [20]. When higher indentation depths are considered, this is reported in the text, always in the validity condition of Hertz model applicability (500 nm and 800 nm for cells measuring up to 15 µm of thickness). To calculate the contact point we used a threshold method [12], keeping the same parameters for all datasets. The analysis was performed using dedicated python software (https://github.com/CellMechLab/nanoindentation (accessed on 2 March 2023), software version: ee04b82) available under an open-source license [12]. At least 50 force-distance curves were acquired for each condition, to achieve statistical relevance.

### 2.4. Cell Morphology Analysis

Digital holography microscopy (DHM) and phase-contrast techniques were used to acquire images of fibroblast cells. DHM is a quantitative phase imaging, label-free technique, used to evaluate single-cell 2D and 3D morphological features [21]. It is based on the interference phenomenon: the sample is illuminated with coherent light and the final image is reconstructed from the interference between a reference beam (unmodified) and the one passing through the sample, which introduces a phase delay. The refractive index of the medium is measured in the setup phase, while the value of the cell refractive index was considered to be 1.38 [22]. Data were acquired using a HoloMonitor M3 digital holography microscope (Phase Holographic Imaging, PHI AB, Lund, Sweden) and analyzed using HoloStudio software (Phase Holographic Imaging PHI AB, Sweden). This allowed us to retrieve the value of the cell thickness. The HoloMonitor M3 is equipped with phase contrast objectives, which were used to distinguish flat and elongated cells.

### 2.5. Statistical Approach

Nanoindentation experiments were carried out in technical triplicates. Outlier removal has been performed in Prism GraphPad through ROUT(Q = 5%) method. Violin plots were used to show the distributions of the results. When needed, average and standard deviation parameters were calculated for the distributions. In order to verify the statistical significance of the results, null hypothesis tests were performed on value distributions. Since data does not satisfy the tests for the normal distribution, the nonparametric Kolmogorov–Smirnov test was applied. *p*-values lower than 0.05 identify statistically robust observations, while higher *p*-values are associated with non-significant (ns) differences between distributions. Dunn’s test was used when multiple comparison analyses were required.

## 3. Results and Discussion

### 3.1. Samples Replication

**Figure 3:**
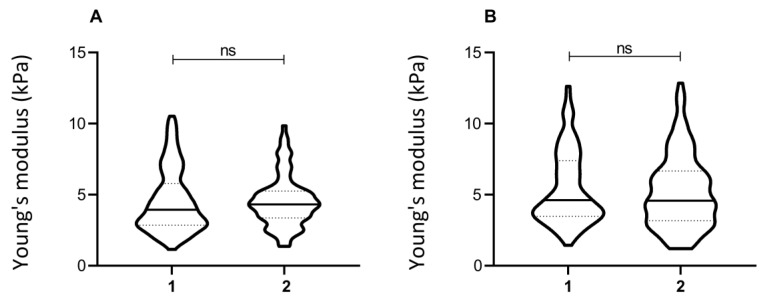
Young’s modulus of technical replicates. Elasticity values were acquired from technical replicates of two different fibroblast lines (line 1, in (**A**), and line 5, in (**B**)) when the acquisition was repeated from different plates (1,2). The violin plot represents the distribution of single-cell Young’s modulus. The non-parametric Kolmogorov–Smirnov test was used to assess the statistical significance.

In order to obtain the data necessary for reliable statistics (at least 50 indentations/condition), often acquisitions are carried out on the same cell culture over different dishes. Such conditions represent the technical replication of samples. In this context we evaluated how the elasticity values varied across the same cell line, plated in different dishes. To this end, cells plated contemporarily in different plates were indented independently. The experiment was repeated using two different cell lines. The distribution of elasticity values for the different conditions is plotted in Figure 3. The statistical difference between the datasets was evaluated as discussed in the methods section, showing that elasticity values are robust across technical replicates, as performed in a standard experimental procedure.

Analogously, it could be necessary to repeat measurements considering biological replicates, i.e., maintaining the same cells and culture conditions but using cells thawed from different cryovials.

**Figure 4:**
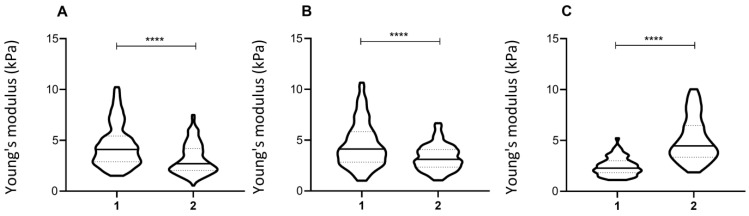
Young’s modulus of biological replicates. The graphs show the distribution of single-cell Young’s modulus, from three different fibroblast lines, each evaluated starting from two different cryotubes (line 1 in panel (**A**), line 2 in panel (**B**), line 3 in panel (**C**)). The non-parametric Kolmogorov–Smirnov test was applied to assess the statistical significance (****p < 0.0001).

We evaluated the impact of using cells derived from different cryovials by testing three different fibroblast cell lines. A statistically significant difference (*p* < 0.001) between the distributions of the Young’s modulus coming from different biological replicates was found (Figure 4). This behavior could be related to the effect of cell freezing and thawing: this could play a crucial role, especially in primary culture, enabling the selection of cell subpopulations or directly altering membrane properties, thus explaining more skewed distributions. Overall, this uncontrolled natural selection, combined with possible differences in cell passage between diverse cryovials, could influence the elasticity of the observed cell culture.

### 3.2. Repetition of the Indentation Site

**Figure 5:**
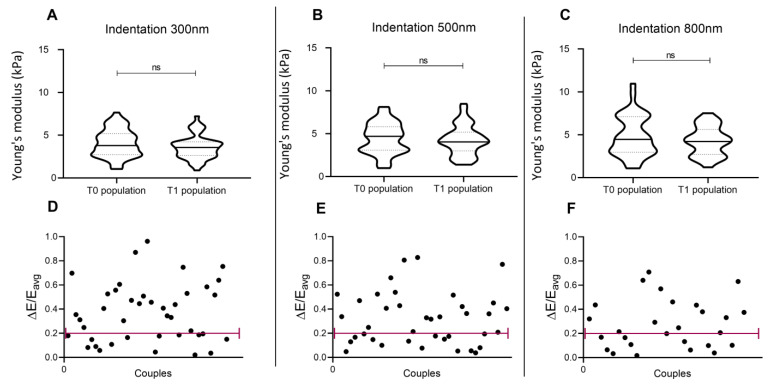
Indentation repeated in the same site of a single cell, 1 min after the first indentation (line 2). Panel (**A**–**C**): Distribution of elasticity values in the cell population, when indentation is performed at 300, 500, and 800 nm. (**D**–**F**): Normalised differences between elasticity values for each cell, obtained from the two consecutive indentations.

In a typical cell mechanics experiment, the operator has to manually choose the indentation site over the cell. An interesting test when measuring cell elasticity consists of the evaluation of whether its value can be reliably and robustly associated with each site of the cell. To monitor this aspect, indentations have been repeated twice, in the same cell site, 60 s apart. The obtained distributions on the cell population do not show significant differences (Figure 5A–C), indicating that the Young’s modulus of the population is rather stable and can be robustly evaluated.

Nevertheless, the comparison between pairs of values retrieved from the same cell showed important dissimilarities. To allow data interpretation, values from two consecutive repetitions differing <20% were considered reproducible. Hypothesizing the impact of indentation depth on the retrieved values, this study has been repeated at 300 nm, 500 nm, and 800 nm indentation. Only 37% (when indenting 300 nm), 44% (when indenting 500 nm), and 48% (when indenting 800 nm) of the measurements were considered reproducible, showing differences in consecutive indentations of the same cell site (Figure 5D–F). This indicates that, at the single-cell level, the repetition of indentation could reveal not always robust results. Such data also show that the indentation depth does not highly interfere with measurements of elasticity repeated in the same cell site.

**Figure 6:**
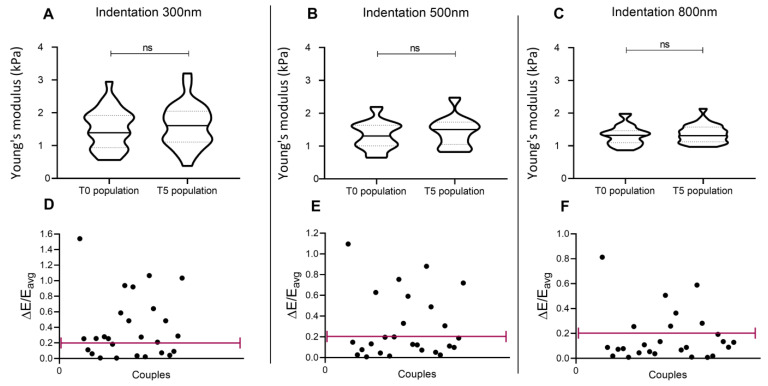
Indentation repeated in the same site of a single cell, 5 min after the first indentation (line 7). Panel (**A**–**C**): Distribution of elasticity values in the cell population, when indentation is performed at 300, 500, and 800 nm. (**D**–**F**): Normalized difference between elasticity values of each cell, obtained from the two consecutive indentations.

In the hypothesis that the differences that emerged when repeating the indentations after 60 s could be related to the process of deformation recovery enabled by the cell after its first indentation, a further set of measurements was performed repeating the indentation 5 min after the first, to increase the cytoskeleton recovery time. Even in this case, the distributions of the cell population do not show significant differences (Figure 6A–C). In this experimental design, a better reproducibility of results was recorded: differences between pairs of values were highlighted to be <20% in 42% of cases when indenting 300 nm, in 65% of cases when indenting 500 nm, and in 73% of cases when indenting 800 nm (Figure 6D–F). This could mean that a longer recovery time partially promotes data reproducibility and highlights that indentation depth can have a different impact on cell recovery.

**Figure 7:**
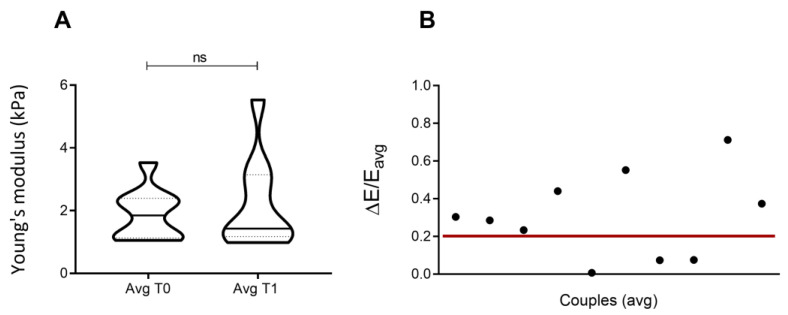
Averaged results from matrix Indentation, 1 min after the first indentation (line 1). (**A**) Distribution of average elasticity values in the cell population (average value from nine indentations per cell, at 300 nm, in different cell regions). (**B**) Normalized difference between elasticity values of each cell, obtained from the two consecutive indentations (average value from nine indentations per cell, at 300 nm, in different cell regions). The statistical analysis relies on the non-parametric Kolmogorov–Smirnov test.

Considering the inhomogeneity of cells, we also acquired elasticity values from different regions of individual cells, to obtain a wider sampling of the studied material and a more exhaustive value to recapitulate cell stiffness values. To this end, two 3 × 3 matrices of 300 nm indentations on a single cell were performed, 60 s apart. This allows us to retrieve nine elasticity values for each cell, at T0 = 0 and T1 = 60 s. Matrix points were 500 nm apart. The protocol was repeated on 10 independent cells from the same cell culture. A single average value was obtained for each cell at T0 and T1, by calculating the mean of the nine stiffness values. Results from the first rounds of indentation were compared to those from the second rounds, both at the population (Figure 7A) and single pair (Figure 7B) levels. The values distribution of the two populations appeared statistically similar (*p*-value = 0.9), whereas when comparing pairs of values (i.e., |E0_avg_ – E1_avg_|/(E0 + E1/2) for each cell), 30% of repeated indentations provided analogous elasticity values for the cell, in line with results shown in Figure 5A.

### 3.3. Cell Shape

**Figure 8:**
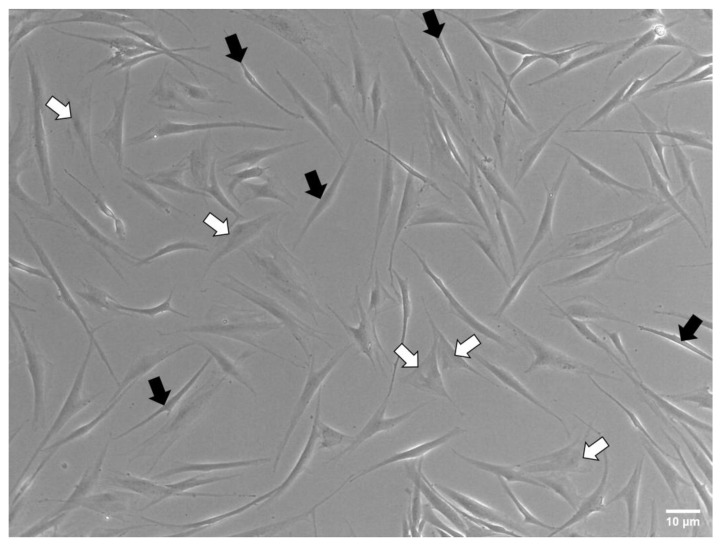
Fibroblast sub-populations. Phase-contrast images highlighted the presence of two different morphological populations: elongated fibroblasts (highlighted with black arrows) and flat fibroblasts (highlighted with white arrows).

Cell lines could sometimes be not homogeneous in shape, especially when primary cultures derived from donors are analyzed, and different sub-populations can emerge. In the primary fibroblast lines used in this work, two subpopulations can be identified in terms of cell shape: one group shows a flat shape, while the other shows elongated morphology (Figure 8).

**Figure 9:**
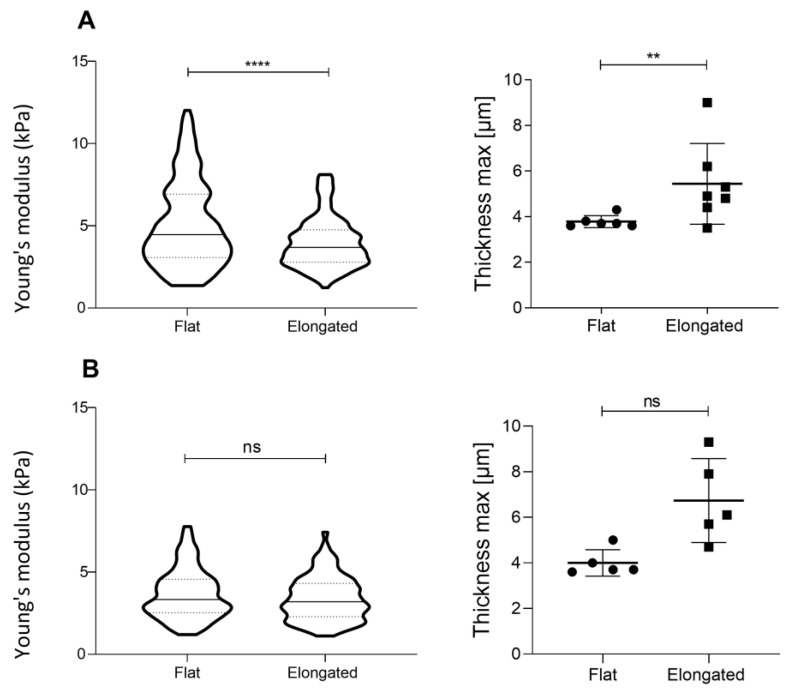
Young’s modulus dependence on cell shape. (**A**) Example of cell line showing differences in cell shape correlating with elasticity values and cell thickness (line 1). (**B**) Example of a cell line where differences in shape do not imply diverse thicknesses, thus showing stable stiffness (line 2). The statistical analysis relies on the non-parametric Kolmogorov–Smirnov test (****p < 0.0001; **p < 0.01).

Cell shape is driven by adhesion structures and cytoskeleton organization, and we expect a mechanical interplay between these two influences to emerge [23]. We performed an experimental test to assess whether different cell shapes correspond to diverse elasticity value ranges. The Young’s modulus distribution obtained from the nanoindentation of lines of fibroblasts where flat and elongated cells appeared, showed that different shapes do not influence cell elasticity. Nevertheless, by coupling stiffness values to cell thickness obtained by holography, a more subtle connection was found between mechanical and morphological features. In particular, when cell shape variation is accompanied by different cell thicknesses (Figure 9A), the elasticity value is impacted, whereas when cell morphology is not associated with variations in thickness (Figure 9B), elasticity values do not demonstrate any relevant changes.

### 3.4. Confluency

**Figure 10:**
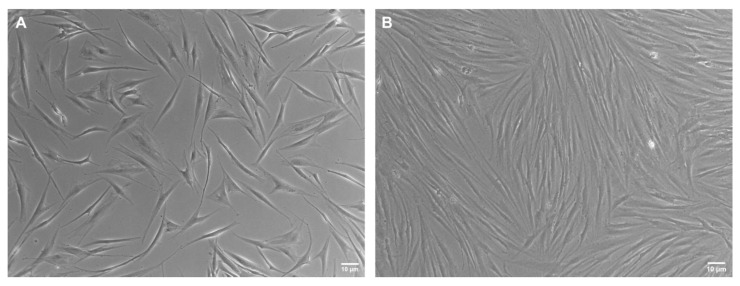
Phase-contrast pictures. Images showing different cell confluence percentages. (**A**) Cells plated with a low percentage of confluency (around 50%) and (**B**) with a high percentage of confluency (around 90%).

**Figure 11:**
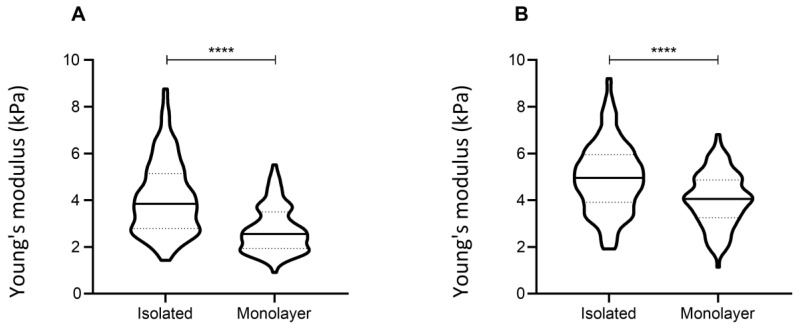
Contribution of cell confluence. Distribution of the single-cell Young’s Modulus, from two different fibroblast lines (lines 2 (**A**) and 4 (**B**)). The statistical analysis is on a non-parametric Kolmogorov–Smirnov test (****p < 0.0001).

Another crucial parameter when designing an indentation experiment is the percentage of confluency of the cell analyzed (Figure 10). High confluence regions produce mechanical cues between adjacent cells that might sensibly impact the measurement of single-cell mechanical properties [24]. Interestingly, we found that cells in monolayers (around 90% confluency) display a strong reduction in Young’s modulus values, compared to the isolated cells at the same passage. This was assessed in two different fibroblast lines (Figure 11).

**Figure 12:**
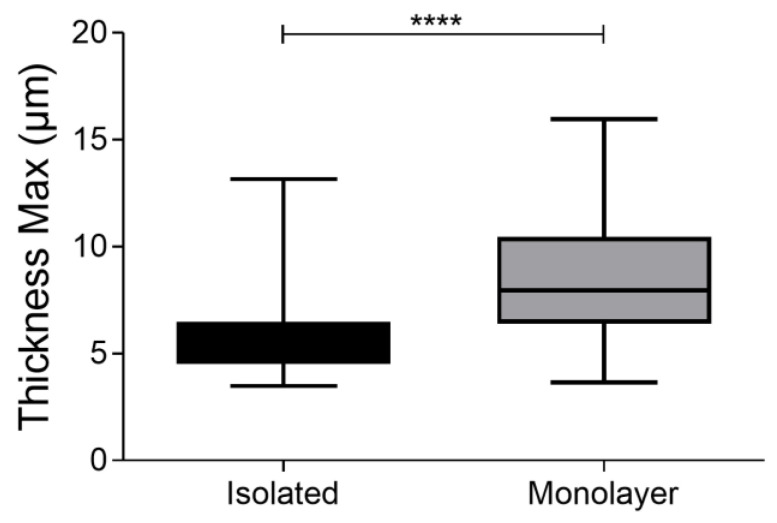
Cell thickness. Cell thickness was calculated by holographic microscopy. The histogram represents the medium value of the thickness of 100 cells compared to 40 islands/monolayers of cells. The statistical analysis is on a non-parametric Kolmogorov–Smirnov test. (****p < 0.001).

When analyzed through holographic microscopy, cells plated with a very high percentage of confluency displayed higher thickness values compared to isolated cells (Figure 12). This information could correlate with lower Young’s moduli. In fact, the literature shows that in monolayer cultures each cell can count on a small adhesion area, which could potentially reflect reduced stiffness [25].

### 3.5. Effect of Starvation on Young’s Modulus Values

It is well recognized that the process of cell growth and division is highly mechanically-driven [26] and that the shape and elasticity of single cells change quite dramatically in different moments of the cycle [27]. To characterize the impact of this biological process, we evaluated the distribution of mechanical properties on cell populations subjected to a starvation protocol (see ‘Materials and methods’). We performed two independent experiments, comparing starved and non-starved cells in the same fibroblast line. Data show statistically significant differences between the two culture conditions.

**Figure 13:**
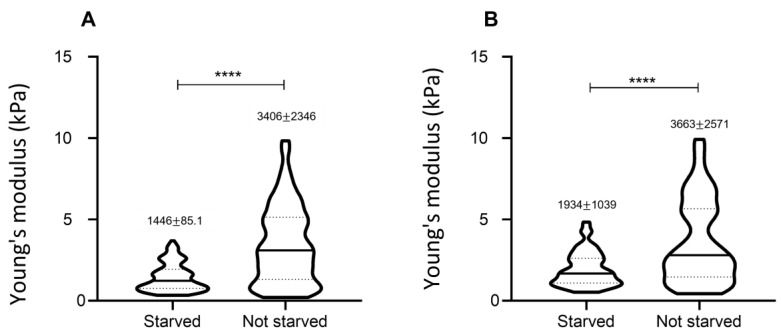
Effect of starvation. Two independent experiments comparing starved and non-starved cells from the same fibroblast line obtained from different cryotubes at the same cell passage (FB line 1, panels (**A**) and (**B**)). Each value of the distribution represents the Young’s Modulus from an individual cell. For each distribution, the average value and the standard deviation are reported. The statistical comparison was performed by means of the non-parametric Kolmogorov–Smirnov test. In both cases, the difference was statistically significant (****p < 0.001).

Starvation seems to cause a reduction in cell data variability (Figure 13), shown by the decrease in data dispersion. As a hypothesis, a possible reason to explain this phenomenon could be that cell starvation synchronizes cells to remain in G0/G1 phase, thus reducing the phase heterogeneity occurring in freely growing cells, which contributes to differences in cell elasticity [28]. Hence synchronization could be responsible for more homogeneous elasticity results.

### 3.6. Number of Passages/Ageing

**Figure 14:**
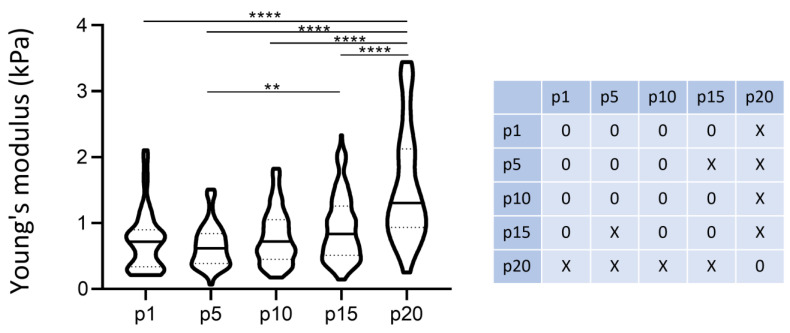
Elastic modulus during cell passages, isolated cells, 300 nm of indentation. The plot shows the distribution of the single-cell Young’s Modulus of fibroblast line 6 during cell passages. The statistical analysis was performed through a Kruscal–Wallis test with Dunn’s test for multiple comparisons correction (**p < 0.01; ****p < 0.001). The matrix reports the cases of significant statistical differences.

To highlight the possible influence of the number of passages on elasticity values, we executed cell stiffness measurements at different numbers of passages. Especially when dealing with primary cultures (as in this case), the number of passages, and related cell age, could be responsible for the variation in many cell parameters, affecting their biological behavior. When dealing with primary cells, which modify rapidly, biological features can change drastically even at a low number of passages. In this case, the elasticity of the cell population was found to change significantly after the 15th passage (Figure 14).

## 4. Conclusions

The mechanical properties of single cells are directly linked to their physiological state since the alteration of traits such as the organization of the cytoskeleton, the maturation of adhesion structures, and the shape of the cell directly impact its viscoelastic properties. There is a huge expectation that cell mechanics will be used, alongside more traditional biochemical markers, to empower the ability in diagnosing and staging life-threatening conditions, such as cancer, and to offer a holistic view of cell phenotype that can enable exciting discoveries in pre-clinical research [29]. Nevertheless, current approaches to measuring cell mechanics still lack the robustness and repeatability required to support this growing field of research. It has been shown that different methods to characterize single-cell mechanics can lead to a spread of results over a few orders of magnitude [13]. This has prompted scientists to favor experimental designs where relative mechanical properties are compared, as obtained with the same device and procedure. In this paper, we adopted this approach and evaluated the impact of the major experimental parameters on single-cell mechanics, measured with a nanoindentation device. Indentations were performed on primary human fibroblast cell lines, evaluating how the determination of Young’s modulus is impacted while repeating the experiment (technical vs. biological replicates and position of the indentation), and how it depends on the shape of the cell (putatively associating strong shape differences to different cell populations), or specific culture conditions (confluency level, cell cycle and starvation, and passaging). We demonstrated that the procedure can be quite insensitive to technical replicates and that the position of indentation might lead to differences on the single-cell level, which would average out on a population basis. Moreover, we clearly observed that several biological conditions can easily lead to divergences in the measured phenotype. Cell confluency, starvation (phase of the cell cycle), and cell passages (aging) significantly impact the results and should be carefully considered and explicitly referred to in nanoindentation experiments. An interesting insight is provided by the dependence of mechanical properties on the shape of the cell. Other than being a confounding factor while characterizing a population, this aspect can offer an opportunity for future works where shape and mechanics are carefully analyzed together.

## Data Availability

Unfiltered data supporting reported results can be found in Zenodo platform at: 10.5281/zenodo.7766179.
